# Sensing of HSV-1 by the cGAS–STING pathway in microglia orchestrates antiviral defence in the CNS

**DOI:** 10.1038/ncomms13348

**Published:** 2016-11-10

**Authors:** Line S. Reinert, Katarína Lopušná, Henriette Winther, Chenglong Sun, Martin K. Thomsen, Ramya Nandakumar, Trine H. Mogensen, Morten Meyer, Christian Vægter, Jens R. Nyengaard, Katherine A. Fitzgerald, Søren R. Paludan

**Affiliations:** 1 Department of Biomedicine, University of Aarhus, Bartholins Allé 6, 8000 Aarhus, Denmark; 2 Aarhus Research Center for Innate Immunology, University of Aarhus, 8000 Aarhus C, Denmark; 3 Department of Molecular Pathogenesis of Viruses, Institute of Virology, Slovak Academy of Sciences, 845 05 Bratislava, Slovak Republic; 4 Department of Infectious Diseases, Aarhus University Hospital Skejby, 8200 Aarhus N, Denmark; 5 Department of Neurobiology Research, Institute of Molecular Medicine, University of Southern Denmark, 5000 Odense C, Denmark; 6 Department of Clinical Medicine, University of Aarhus, 8200 Aarhus N, Denmark; 7 Division of Infectious Diseases and Immunology, Department of Medicine, University of Massachusetts Medical School, Worcester, Massachusetts 01605, USA

## Abstract

Herpes simplex encephalitis (HSE) is the most common form of acute viral encephalitis in industrialized countries. Type I interferon (IFN) is important for control of herpes simplex virus (HSV-1) in the central nervous system (CNS). Here we show that microglia are the main source of HSV-induced type I IFN expression in CNS cells and these cytokines are induced in a cGAS–STING-dependent manner. Consistently, mice defective in cGAS or STING are highly susceptible to acute HSE. Although STING is redundant for cell-autonomous antiviral resistance in astrocytes and neurons, viral replication is strongly increased in neurons in STING-deficient mice. Interestingly, HSV-infected microglia confer STING-dependent antiviral activities in neurons and prime type I IFN production in astrocytes through the TLR3 pathway. Thus, sensing of HSV-1 infection in the CNS by microglia through the cGAS–STING pathway orchestrates an antiviral program that includes type I IFNs and immune-priming of other cell types.

## Abstract (web summary)

The cGAS–STING pathway is an important immune defence pathway against viral infection, including HSV-1. Here the authors use an HSV-1 encephalitis model and show microglia are the main producers of type 1 interferons that induce antiviral activity in neurons and prime the TLR3-interferon pathway in astrocytes.

## Graphical abstract

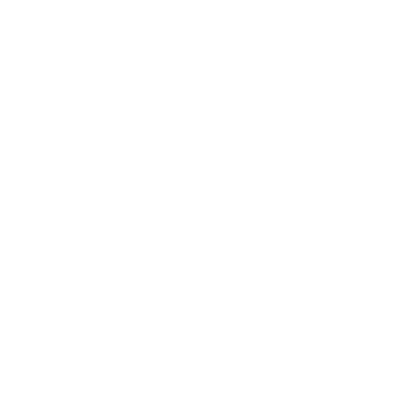

Viral infections in the central nervous system (CNS) can result in devastating diseases such as encephalitis, which can cause irreversible damage to the CNS and fatality^1^. Herpes simplex virus (HSV) type 1 is a neurotropic virus and is a major cause of CNS infections, including herpes simplex encephalitis (HSE)^1^. The innate immune response to infection is essential for host control of HSV^2^. The innate immune system utilizes pattern recognition receptors (PRRs) to detect microbial molecules and stimulate host defense, in which type I interferon (IFN) have a particularly important role in viral infection^3^. Viruses induce type I IFN expression mainly through nucleic acids and (RNA-/DNA-sensing) PRRs, as well as downstream type I IFN-inducing signalling pathways, which are well described^4^,^5^.

Genetic studies in humans have led to the identification of defects in the RNA-sensing TLR3 pathway in patients with HSE^6^,^7^,^8^,^9^,^10^, and this finding has been confirmed in a mouse model for HSV-2 myelitis, in which astrocytes sense the virus in a TLR3-dependent manner^11^. However, another study using induced pluripotent stem cells derived from patient dermal fibroblasts suggested that neurons and oligodendrocytes control HSV-1 replication in a TLR3-dependent manner *in vitro*^12^. Thus, the cell-type-specific action of the TLR3 pathway is context dependent and it remains unresolved whether a priming step is needed and also whether TLR3 acts in concert with other innate pathways to exert antiviral activity in the CNS.

HSV-1 carries its genetic information in the form of double-stranded DNA (dsDNA), and advances in the basic understanding of innate immune sensing have revealed that cytosolic dsDNA is a potent inducer of innate immune responses^13^. Several DNA sensors have been identified, including cyclic GMP–AMP synthase (cGAS)^14^, and all type I IFN-inducing cytoplasmic DNA sensors signal through the adaptor protein stimulator of type I IFN genes (STING)^13^. We reported that HSV-1 entry into macrophages leads to abundant accumulation of viral DNA in the cytoplasm, which is where the DNA is sensed to induce type I IFN expression^15^. Microglia are resident macrophages of the CNS and have important roles in both protective and pathological immune responses in the CNS^16^. Microglia express a wide panel of PRRs and are importance for defense against HSV-1 (ref. 17). Despite the well-documented role for DNA sensing in recognition of several pathogens, published data on the mechanisms of action of this pathway *in vivo* are limited. For instance, it is unknown which cell types utilize the STING-dependent DNA-sensing pathway to recognize HSV-1 *in vivo* and how this may influence the intercellular communication network in the tissue.

In this work, we demonstrate that microglia are the main producers of type I IFN after HSV-1 infection and this cytokine production is STING dependent. cGAS deficiency and STING deficiency renders mice susceptible to HSE after peripheral infection in the eye, and correlates with impaired type I IFN expression in the CNS. *In vitro* infection of neurons and glia cells reveals that microglia, but not neurons or astrocytes, exhibit increased viral replication in the absence of STING. By contrast, *in vivo* STING deficiency increases HSV-1 replication in microglia. Interestingly, HSV-1-infected microglia transfer antiviral activity to neurons in a STING-dependent manner. In addition, treatment of astrocytes with type I IFN or supernatants from infected wild type (WT), but not STING deficient, microglia induces TLR3 expression by astrocytes, thus enabling a response through these pathways. Collectively, these data suggest that innate sensing of HSV-1 DNA in the brain by microglia induces a paracrine antiviral type I IFN response and also enables innate sensing pathways in other cell types, including the TLR3 pathway known to be essential for protection against HSE in humans.

## Results

### Mice deficient in cGAS or STING are susceptible to HSE

**Figure 1:**
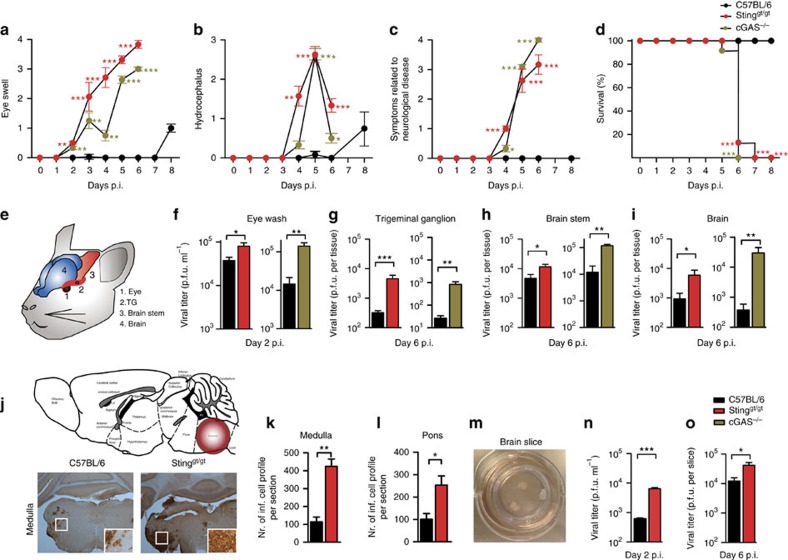
Mice deficient in cGAS or STING are susceptible to HSE and exhibit impaired antiviral responses. Mice were infected in the cornea with 1 × 10^6^ PFU per eye of HSV-1 (strain Mckrae). On subsequent days, animals were scored for (**a**) eye swelling, (**b**) hydrocephalus, and (**c**) symptoms related to neurological disease and (**d**) survival. (**e**) Route of virus spread from the eye to the CNS. (**f**–**i**) Eye washes, trigeminal ganglia, brain stem and brains were isolated on the indicated time points post infection, and viral load was quantified using plaque assay. *n*=9 mice per group (**a**–**i**). (**j**) Tissue section from the brain stem of WT and Sting^gt/gt^ mice infected for 6 days with HSV-1 were stained with anti-HSV-1. *n*=5-6 mice per group. The original magnifications are 2.5 × and 20 × for the zoomed in images. (**k**–**l**) The number of HSV-1-positive cells in six tissue sections from the medulla and pons were quantified, and presented as means ± s.e.m. *n*=3–9 per group. (**m**–**o**) Organotypic brain slices from WT and Sting^gt/gt^ mice were cultured and infected with 5 × 10^3^ PFU of HSV-1. The viral load in (**n**) the culture medium on day 2 and (**o**) in homogenized brain slices on 6 days post infection was determined by plaque assay. Data are shown as mean values ± s.e.m., *n*=6–8 wells with three brain slices in each. Symbols for *P*-values used in the figures: *0.01<*P*<0.05; **0.001<*P*<0.01; ***P<0.001; NS, not significant. Red and green asterisks indicate *P*-values between WT and relevant KO mice at specific days post infection.

To mimic the natural route of HSV-1 entry into the CNS through retrograde transport we used a model for ocular HSV-1 infection. For this study, we used cGAS^−/−^ and Goldenticket (gt) mice, which harbour a single nucleotide variant (T596A) of STING that functions as a null allele^18^. By western blotting, we demonstrated the lack of STING expression in the Gt mice, including in the CNS (Supplementary Fig. 1). When WT, cGAS^−/−^ and Sting^gt/gt^ mice were challenged with HSV-1 (strains Mckrae or KOS), we observed severe disease development in mice with a defective cGAS–STING pathway, as shown by disease scores reflecting infection in both the eye (Fig. 1a; Supplementary Fig. 2a,c) and in the CNS (Fig. 1b,c, Supplementary Fig. 2d). The mice lacking STING also lost weight rapidly after infection with the Mckrae strain (Supplementary Fig. 2e), and succumbed to infection on day 6 post infection whereas the WT mice survived through the 8 days of the experiment (Fig. 1d). Previous reports have demonstrated elevated HSV-1 load in the cornea of STING-deficient mice after HSV-1 infection^19^,^20^. When examining viral titres at different locations from the site of inoculation to the CNS (Fig. 1e), we were able to detect HSV-1 (Mckrae) in all compartments and consistently found higher viral load in samples from the cGAS- and STING-deficient mice (Fig. 1f–i). Further analysis of the early events in HSV-1 replication in neuronal tissue showed that the levels of HSV-1 in trigeminal ganglia was comparable between WT and STING^gt/gt^ mice at day 2 post infection, after which we observed significantly elevated viral load in trigeminal ganglia from STING-deficient mice (Supplementary Fig. 2f). While HSV-1 was detectable in the brain stem from day 3 after infection in WT mice, the virus reached the brain stem already on day 2 in STING-deficient mice, and was detected in higher levels than in WT mice from the time of entry into the brain (Supplementary Fig. 2g). Infection with the HSV-1 KOS strain also led to elevated viral load in the brain stem in the STING-deficient mice, but this strain was unable to spread further in the brains of STING-deficient mice, similar to the WT mice (Supplementary Fig. 2h,i).

To evaluate whether a defective cGAS–STING pathway affects the path of HSV-1 neuro-entry and the pattern of viral spread within the CNS, we sliced whole mouse brains from infected mice into 60 μm sections and stained every sixth section with anti-HSV-1 antibodies. Areas positive for HSV-1 were observed in the medulla of brains from both WT and STING-deficient mice, although we observed a larger number of HSV-1-infected cells in the sections from the STING-deficient mice (Fig. 1j–l). These data are consistent with HSV-1 entering into the CNS through the neuronal route via the trigeminal nerve. When looking beyond the medulla, we found HSV-1-positive areas only in the pons in WT mice (Fig. 1k; Supplementary Fig. 3). By contrast, in the brains from STING-deficient mice, the virus was not restricted to the area around the medulla, but was widely disseminated to the midbrain, hypothalamus, and the preoptic area (Supplementary Fig. 3).

Since we observed higher viral load in cGAS- and STING-deficient mice already in the eye, and also observed accelerated viral entry in the brain tissue, it remained possible that the elevated viral load in the CNS was due to a requirement of the cGAS–STING pathway in the periphery and not within the brain. Therefore, we isolated and cultured brain slices from newborn WT and STING-deficient mice, and infected with HSV-1 (Fig. 1m–o). The homogenized slices, as well as supernatants isolated from cultures of STING-deficient brains contained significantly more virus than that of WT brain slices, thus suggesting that the brain *per se* relies on STING to mount a defense against HSV-1 infection.

### Elevated viral load in STING-deficient microglia and neurons

**Figure 2:**
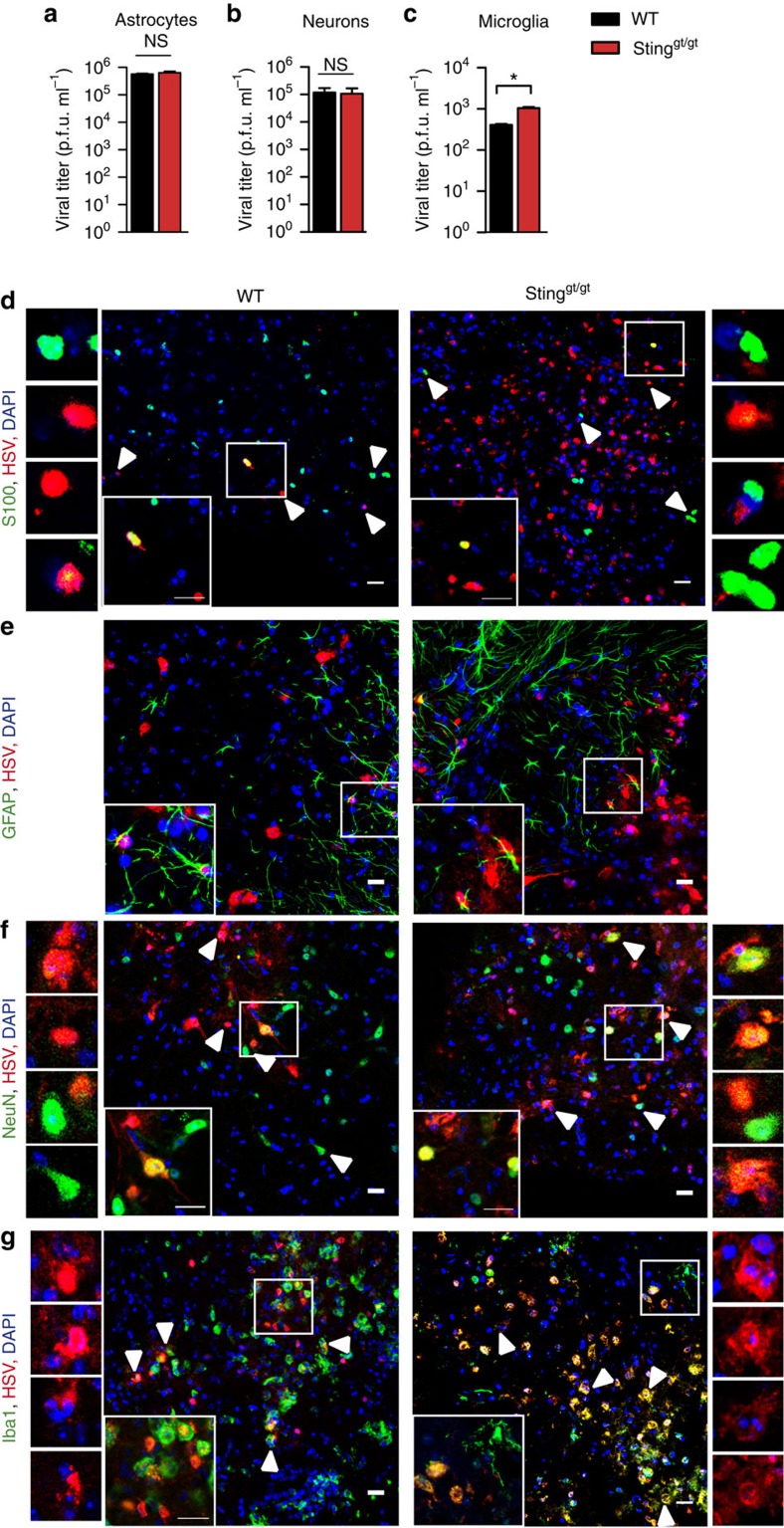
STING is essential for restriction of HSV-1 in microglia in vitro and for antiviral control in neurons *in vivo*. (**a**–**c**) Astrocytes, neurons and microglia from WT and Sting^gt/gt^ mice were cultured *in vitro* and infected with HSV-1 (MOI 1). Supernatants were collected 48 h later and virus was quantified by plaque assay. Data are presented as means ± s.e.m. *0.01<*P*<0.05; NS, not significant, *n*=5–8 per group. (**d**–**g**) Tissue sections from the brain stem of six WT and 6 Sting^gt/gt^ mice isolated 6 days after infection with HSV-1 (1 × 10^6^ PFU per eye) were stained with an antibody against HSV-1 and antibodies against cell-type-specific markers: (**d**) S100, a nuclear astrocyte marker; (**e**) GFAP, a fibrillary astrocyte marker; (**f**) NeuN, neurons; and (**g**) Iba1, microglia. Scale bar, 20 μm. Cells marked by arrow-heads are magnified in the images to the left and right of the large images in **d**,**f** and **g**. The magnified images in **g** show staining for HSV-1 and DAPI without the cell-type-specific marker (Iba1).

To examine the role for STING in cell-autonomous control of HSV-1 replication, we isolated neuronal cells from newborn mice and differentiated cells *in vitro* into neurons, astrocytes and microglia. The purity of the specific cell types was evaluated to be in the range 89–97% depending on the cell type (Supplementary Fig. 4). Consistent with the literature on HSV-1 replication in human, in induced pluripotent stem cell-derived neuronal cells^12^, HSV-1 replicated very well in WT neurons and astrocytes, but only weakly in microglia (Fig. 2a–c). In neurons and astrocytes, STING deficiency did not lead to elevated viral growth. In microglia, STING deficiency was associated with the elevated virus yield (Fig. 2c), although the levels of virus were 3–4 logs lower than in neurons and astrocytes. These data suggest that STING is involved in control of HSV-1 replication in microglia, whereas astrocytes and neurons utilize antiviral mechanisms independent of STING *in vitro*.

To examine which cell types harboured viral antigens in the CNS of WT and STING-deficient mice, tissue sections from brain stems were stained with antibodies against HSV-1 and cell-type-specific markers. As expected, we observed a larger proportion of HSV-1-positive cells in the STING-deficient mice as compared with WT mice (Fig. 2d–g). The staining for HSV-1 antigens exhibited a preferential nuclear pattern consistent with HSV-1 replication occurring in the nucleus (Fig. 2d). However, we did not observe a higher number of astrocytes containing viral antigen in the brains from STING-deficient mice as compared with WT mice (Fig. 2d,e), consistent with the findings *in vitro* (Fig. 2a). By contrast, when examining viral antigens in neurons *in situ*, we observed many more infected Neuronal Nuclei (NeuN)-positive cells in STING-deficient versus WT mice (Fig. 2f). Quantification of the immunohistochemistry data showed that 12.4±2.6% of S100^+^ WT cells (astrocytes) were HSV-1^+^, while 14.9±3.2% of the STING-deficient astrocytes were HSV-1^+^. For the neurons, 9.9±7.7% of WT NeuN^+^ cells were positive for HSV-1, while 33.7±1.5% of the STING-deficient NeuN^+^ cells were HSV-1^+^. With respect to microglia, the data showed a pronounced elevation in the level of viral antigen in cells from STING-deficient mice as compared with WT mice (Fig. 2g). However, the viral antigen in microglia mainly localized to the cytoplasm and not the nucleus, suggesting uptake of viral antigen rather than the productive replication. These data are consistent with the *in vitro* data showing only modest HSV-1 replication in microglia cells, although this was significantly elevated in the absence of STING (Fig. 2c).

Altogether, the elevated viral load in STING-deficient neurons *in vivo* but not *in vitro* suggests the existence of a STING-dependent intercellular network, which transfers antiviral signals to neurons *in vivo*.

### STING-dependent type I IFN production by microglia

**Figure 3:**
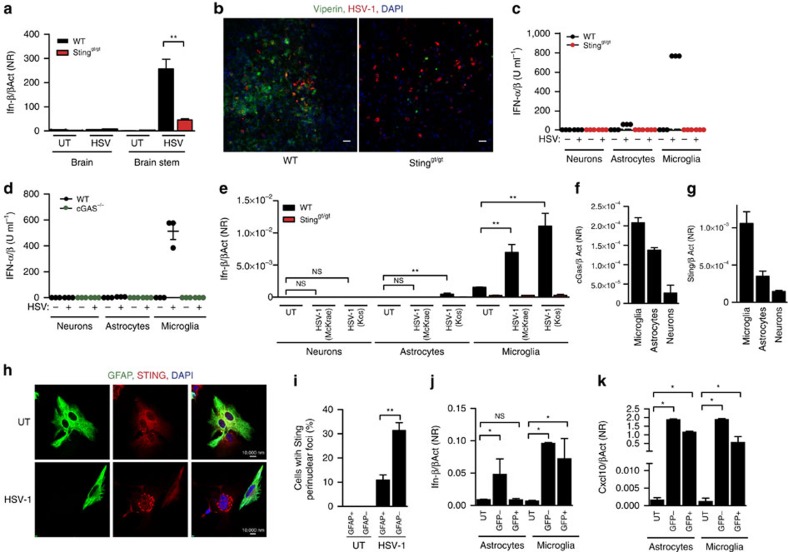
Microglia utilize the cGAS–STING pathway to mount strong IFN responses to HSV-1 infection. (**a**) RNA from brain stem of WT and Sting^gt/gt^ mice infected for 6 days with HSV-1 (1 × 10^6^ PFU per eye) or media alone (UT) were analysed by RT–qPCR for levels of Ifn-β mRNA. (**b**) Tissue sections of brain stems from WT and Sting^gt/gt^ mice infected for 6 days with HSV-1 were stained with antibodies against viperin and HSV-1. *n*=5–6 mice per group. (**c**–**e**) Astrocytes, neurons and microglia from WT, cGas^−/−^ and Sting^gt/gt^ mice were cultured *in vitro* and infected with HSV-1 (MOI 1). Supernatants or total RNA were collected 24 and 6 h later, respectively. The supernatants were assayed for type I IFN bioactivity, and the RNA was analysed for IFN-β mRNA levels. (**c**,**d**) Data are representative of three repeats and are presented as individual measurements. (**f**,**g**) Total RNA from purified microglia, astrocytes, and neurons were analysed for expression of cGAS and STING. (**h**,**i**) Isolated astrocytes and microglia were mixed and infected with HSV-1. The cells were fixed 4 h later and stained with antibodies against GFAP and STING. (**i**) Cells with translocation of STING from diffuse to perinuclear foci staining patterns were quantified in GFAP^+^ (astrocytes) and GFAP^−^ (microglia) and presented as means of eight measurements ± s.e.m. (**j**,**k**) Mixed cultures of astrocytes and microglia were infected with HSV-1-expressing eGFP driven by the CMV promoter (MOI 3). 6 h later, the cells were sorted into GFP^+^ and GFP^−^ populations, and further sorted into astrocytes and microglia. Total RNA from the four populations was analysed together with uninfected controls for expression of IFN-β and CXCL10. UT, GFP-negative population originating from uninfected mixed cultures. All RT–qPCR data in this figure were normalized to β-actin and presented as means ± s.e.m. *n*=5–8 per group Symbols for *P*-values used in the figures: *0.01<*P*<0.05; **0.001<*P*<0.01; ***P<0.001; NS, not significant.

The data presented above suggest that microglia rely on STING to activate antiviral defense, and also that there is STING-dependent transfer of antiviral signals through the tissue in the CNS. Type I IFNs are pivotal for control of HSV-1 replication in the CNS, and we therefore wanted to evaluate the expression and function of this class of cytokines in the brain during HSV-1 infection. In brain stem homogenates from infected mice, significantly reduced induction of IFN-β gene expression was observed after HSV-1 infection in mice lacking STING (Fig. 3a). This led to the reduced expression of the IFN-stimulated gene (ISG) viperin in the infected tissue in STING-deficient mice (Fig. 3b). Although viperin has been shown not to have antiviral activity against WT HSV-1 *in vitro*^21^, we used it as a marker of ISG expression.

To identify which cells of the CNS produced type I IFN, we isolated supernatants from neurons, astrocytes and microglia infected with HSV-1 and measured type I IFN bioactivity. While neurons failed to induce type I IFN in response to HSV-1 infection, astrocytes mounted a clear but modest IFN-α/β production (Fig. 3c). In contrast to this, microglia mounted response to HSV-1 infection with production of high levels of type I IFN, and this was entirely dependent on STING and cGAS (Fig. 3c,d). Similar data were obtained when we examined IFN-β expression at the messenger RNA (mRNA) levels in the three cell types with both McKrae strain and with the KOS strain (Fig. 3e).

Given the finding that microglia responded to HSV-1 infection with the most potent type I IFN response in a STING-dependent manner, we wanted to evaluate whether the cGAS–STING pathway was more active in this cell type than in astrocytes and neurons. To test this, we first determined the expression levels of cGAS and STING in neurons, astrocytes, and microglia, and observed that the ability of the cell types tested to produce type I IFN correlated with the expression of cGAS and STING (Fig. 3f,g). In line with this, and mirroring what was observed after HSV-1 infection, the relative stimulation of type I IFN production by synthetic DNA in the three cell types was neurons<astrocytes<microglia (Supplementary Fig. 5). On DNA stimulation, STING translocates to vesicular structures^22^, and accumulation of perinuclear STING foci is often used as a measure of activation of the STING pathway. To further examine whether the cGAS–STING pathway is activated more potently in microglia than astrocytes, we mixed these two cell types, infected with HSV-1, and used formation of STING foci as readout. In line with the stronger expression of type I IFNs by microglia cells, we observed formation of STING foci in a significantly higher proportion of the microglia (glial fibrillary acidic protein (GFAP)^−^) as compared with the astrocytes (GFAP^+^; Fig. 3h,i).

HSV-1 can enter most cell types, but only replicates efficiently in a subset of cells. The data shown in Fig. 2a,c demonstrate that among the identified type I IFN-producing cells, HSV-1 replicates efficiently in neurons and astrocytes but less so in microglia. Productive viral replication may impair various cellular functions, including production and responsiveness to type I IFN. Therefore, we wanted to evaluate whether the establishment of productive replication affected the ability of the different cell types to express IFN-β. For this purpose, we infected cells with a high multiplicity of infection (MOI) of HSV-1 virus expressing enhanced green fluorescent protein (eGFP) under the control of a constitutive promoter, and sorted GFP^+^ and GFP^−^ cells for further analysis. As shown in Supplementary Fig. 6a, expression of GFP correlated with the expression of the late viral protein gB, thus validating that GFP expression was a reasonable measure for productive HSV-1 replication. For the astrocytes, we found that GFP− cells were stimulated to express IFN-β, whereas the productively infected astrocytes were unable to produce IFN-β (Fig. 3j). By contrast, microglia produced IFN-β in response to HSV-1 infection irrespective of whether the virus had established productive infection or not (Fig. 3j). When examining expression of ISGs, we found that astrocytes produced this class of genes even when productively infected (Fig. 3k; Supplementary Fig. 6b,c). This was seen over a range of infectious doses (Fig. 3j,k; Supplementary Fig. 6d,e). This suggests that although the productive viral replication impaires production of IFN-β in astrocytes, the ability to respond to type I IFNs is retained.

Thus, microglia are the main source of type I IFN, during HSV-1 infection of CNS cells and evokes this response in a STING-dependent manner, which is not affected by the establishment of productive infection by the virus.

### STING-independent recruitment of microglia to infection foci

**Figure 4:**
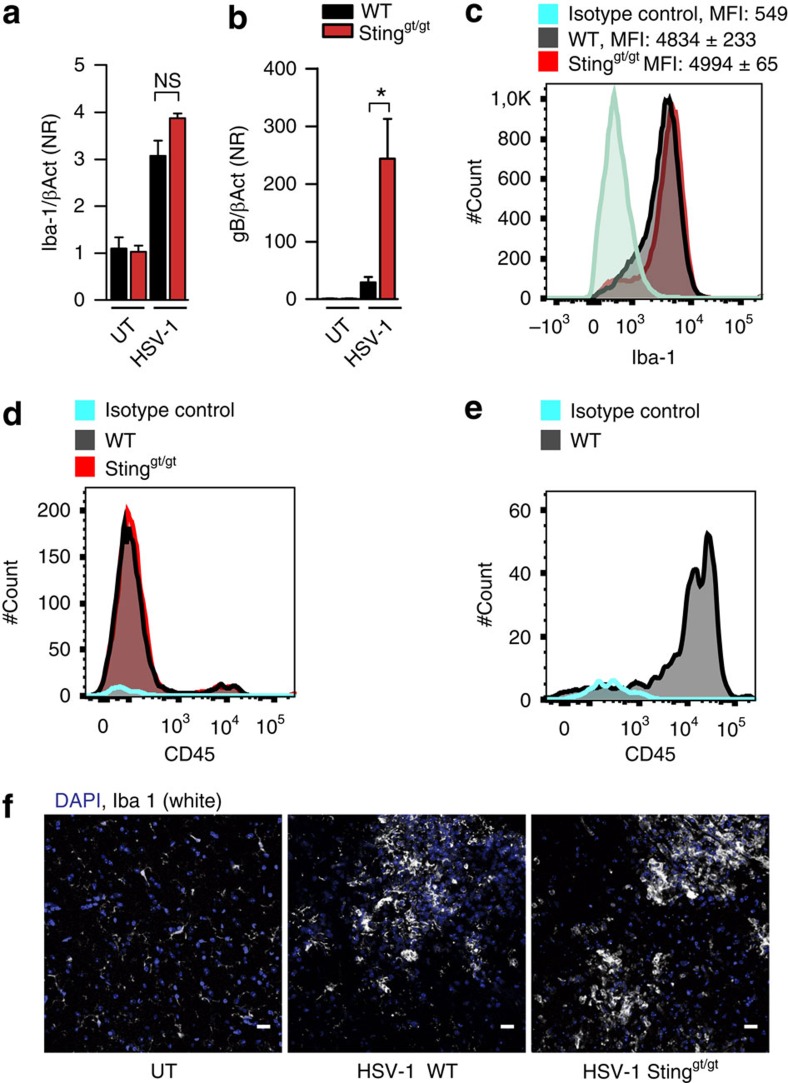
Microglia accumulate at sites of infection in the CNS in a STING-independent manner. (**a**,**b**) RNA from brain stem of WT and Sting^gt/gt^ mice infected for 6 days with either HSV-1 (1 × 10^6^ PFU per eye) or media alone (UT) were analysed by RT–qPCR for levels of Iba1 and gB mRNA. The data were normalized to β-actin and are presented as (means ± s.e.m.) fold induction relative to the WT UT. *n*=3–5 per group. (**c**,**d**) Single cells isolated from brain stems of WT and Sting^gt/gt^ mice infected for 6 days with HSV-1 were analysed by flow cytometry for expression of (**c**) Iba-1 or (**d**) CD45 staining of CD11b^+^ sub-gated cells. Data from representative mice from each group is shown together with median fluorescence intensity (MFI) ±s.d. *n*=6 per group (**e**) as control for CD45^hi^ staining, CD45 staining of CD11b^+^ sub-gated splenocytes was performed on a non-infected mouse. (**f**) Tissue sections of brain stems from WT and Sting^gt/gt^ mice infected for 6 days with HSV-1 or media alone (UT) were stained with an antibody against Iba1, *n*=4–5 per group, Scale bar, 20 μm.

Next, we were interested in evaluating the STING-dependent nature of the accumulation of microglia and expression of type I IFN-stimulated genes in the foci of infection in the brain. Therefore, we first isolated RNA from the brain stems of WT and STING-deficient mice infected for 6 days. As seen in Fig. 4a, brain stems of infected mice had higher levels of a microglia marker (Iba1) mRNA than uninfected mice, and this was independent of the genotype of the mice, despite significantly higher levels of viral transcripts in the brain stem of the STING-deficient mice (Fig. 4b,c). Since the elevation of Iba1 mRNA levels could be caused by either induction of the gene or recruitment or division of Iba1-expressing cells, we also isolated single cells from brain stems of infected WT and STING-deficient mice to perform flow cytometric analysis for Iba1. This revealed comparable results from the two sets of mice, both in terms of cell counts and intensity of the fluorescent signal (Fig. 4d). Since Iba1 can be expressed by cell types other than microglia, such as bone-marrow-derived macrophages, we determined the cell population expressing CD11b^+^CD45^lo-Medium^ to define microglia. This analysis showed comparable number of CD11b^+^CD45^lo-Medium^ in the brain stem of HSV-1-infected WT and STING-deficient mice (Fig. 4e; Supplementary Fig. 7a,b). Moreover, these data demonstrate that <2% of the gated single cells in the infected brain stem are CD11b^+^CD45^high^ as compared with >15% being CD11b^+^CD45^lo-Medium^ (Supplementary Fig. 7b,c). This suggests that microglia, rather than recruited macrophages, are accumulating in infected areas at the time point examined. Finally, we stained brain stem tissue sections from untreated and HSV-1-infected mice with anti-Iba1 and analysed microscopically. Using this approach, we easily observed microglia in the infected areas with no noticeable difference between the genotypes (Fig. 4g). Thus, accumulation of microglia in areas of HSV-1 infection in the brain occurs in a manner independent of STING.

**Figure 5:**
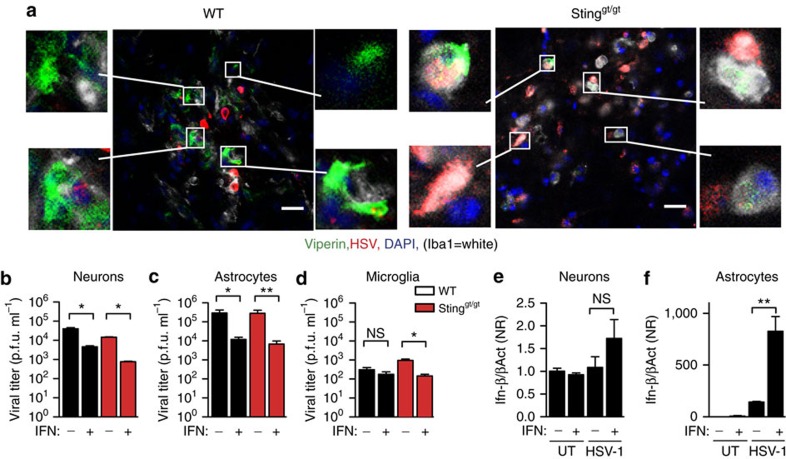
Dissemination of the IFN response in the infected brain depends on STING. (**a**) Tissue sections from the brain stem of WT and Sting^gt/gt^ mice infected for 6 days with HSV-1 (1 × 10^6^ PFU per eye) were stained with an antibody against viperin, HSV-1 and Iba1 (microglia), *n*=4–6 mice per group. Scale bar, 20 μm. Boxed cells are magnified in the images to the left and right of the large images. (**b**–**d**) Astrocytes, neurons and microglia from WT and Sting^gt/gt^ mice were cultured *in vitro*, treated with IFN-α/β (25 U ml^−1^) and infected with HSV-1 (MOI 1). Supernatants were isolated 48 h later, and virus yield was measured by plaque assay. Data are presented as means ± s.e.m., *n*=5–8. (**e**,**f**) Neurons and astrocytes were cultured *in vitro* and treated with IFN-α/β (25 U ml^−1^) and infected with HSV-1 (MOI 1). Total RNA was isolated 6 h later and analysed for IFN-β mRNA levels. Data are normalized to β-actin levels and are presented as (means ± s.e.m.) fold induction relative to the WT UT, *n*=5–8 per group Symbols for *P*-values used in the figures: *0.01<*P*<0.05; **0.001<*P*<0.01; ***P<0.001; NS, not significant.

Next, we wanted to examine the role of the STING pathway in mediating the type I IFN response in cell types other than microglia in and around the infected area in the CNS. Tissue sections from the brain stem of uninfected and HSV-1-infected WT and STING-deficient mice were stained with anti-viperin, anti-HSV-1 and anti-Iba1 antibodies and analysed by confocal microscopy. First, we noted that viperin^+^ staining, consistent with the data shown in Fig. 3b found in many WT cells was largely absent in HSV-1^+^ cells, suggesting that type I IFN stimulation did prevent the efficient viral replication in the brain (Fig. 5a). Second, we found viperin-positive staining in both Iba1^+^ and Iba1^−^ cells in WT mice, thus suggesting stimulation of type I IFN responses also in non-microglial cells. Interestingly, in sections from STING-deficient mice, the modest positive staining for viperin was seen preferentially in microglia with 92±3% of viperin^+^ cells also being Iba1^+^. These data suggest that STING is essential to evoke expression of ISGs in non-microglial cells in the CNS.

To start elucidating how IFNs may functionally modulate non-microglial cells, astrocytes and neurons were treated with IFN-α/β, followed by infection with HSV-1. Subsequently, we evaluated the ability of these cell types to control infection and induce IFN-β gene expression. Type I IFN treatment led to significantly lower virus replication in both cell types, independent of the genotype of the infected cell (Fig. 5b–d). Moreover, although type I IFN treatment did not enable neurons to express IFN-β on HSV-1 infection, this treatment primed astrocytes to become high IFN-β producers in response to HSV-1 infection (Fig. 5e,f). Thus, paracrine stimulation of neurons and astrocytes with type I IFN evokes antiviral activity in both cell types and primes virus-induced IFN-β expression in astrocytes.

### Microglia induce STING-dependent antiviral programs

**Figure 6:**
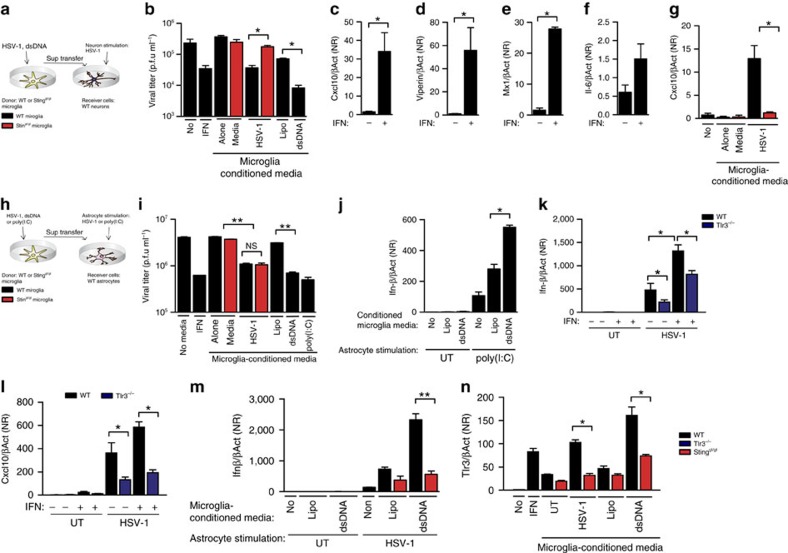
Microglia induce STING dependent antiviral programs in neurons and prime the TLR3 pathway in astrocytes. (**a**,**h**) Illustration of a cellular model used to study intercellular communication between cells of the CNS. Supernatants from WT or Sting^gt/gt^ microglia cultures treated for 24 h with HSV-1 (MOI 1), Lipofectamine, dsDNA (2 μg ml^−1^) or poly(I:C) (5 μg ml^−1^) were ultraviolet-inactivated and transferred to (**b**,**g**) neuron and (**i**,**j**,**m**,**n**) astrocyte cultures. Some cultures received IFN-α/β (25 U ml^−1^) or medium alone. After pretreatmet with microglia supernatants or IFN-α/β for 17 h (**b**,**i**) the neuron and astrocyte cultures were infected with HSV-1 (MOI 1). Supernatants were harvested 48 h later and virus yield was measured by plaque assay. (**c**–**g**) Total RNA from neuron cultures stimulated with IFN-α/β (25 U ml^−1^) for 17 h was analysed for expression of (**c**–**e**) the ISGs Cxcl10, viperin, Mx1 and (**f**) the inflammatory cytokine Il6. (**g**) Total RNA from neuron cultures stimulated with conditioned media from untreated or HSV-1-infected microglia was analysed for expression of Cxcl10 by RT–qPCR. (**j**) Astrocytes pretreated with the indicated conditioned microglia media were stimulated with extracellular poly(I:C) (5 μg ml^−1^) for 6 h, total RNA was isolated and Ifnβ mRNA was measured. (**k**,**l**) WT and TLR3^−/−^ astrocyte cultures were treated with IFN-α/β (25 U ml^−1^) and infected with HSV-1 (MOI 1). Total RNA was isolated 6 h later and analysed for levels of Ifn-β and Cxcl10 mRNA. (**m**) Supernatants from WT and Sting^gt/gt^ microglia cultures were transferred to WT astrocytes, which were stimulated with HSV-1 (MOI 1) for 6 h. Total RNA was isolated and levels of Ifn-β were measured. (**n**) The WT astrocytes were stimulated with IFN-α/β (25 U ml^−1^) or supernatants from microglia stimulated with dsDNA or HSV-1, 6 h after the TLR3 mRNA were measured. Data are presented as means ±s.e.m. All RT–qPCR data in this figure were normalized to β-actin levels and are presented as (means ± s.e.m.) fold induction relative to the WT UT. Symbols for *P*-values used in the figures: *0.01<*P*<0.05; **0.001<*P*<0.01; ***P<0.001; NS, not significant, *n*=5–8 per group.

Finally, we were interested in determining whether microglia were able to deliver signals to other cell types in the CNS and to evaluate the role of STING in these events. To do this, we used a model where conditioned medium from microglia were transferred to cultures of neurons or astrocytes, and functional analyses were performed in these cells (Fig. 6a,h). First, we collected media from WT and STING-deficient microglia challenged with HSV-1 or synthetic DNA and added to neuron cultures. For comparison, we also treated neurons directly with IFN-α/β. As expected, type I IFN and supernatants from dsDNA-treated microglia significantly impaired HSV-1 replication in neurons (Fig. 6b). Importantly, supernatants from HSV-1-infected WT microglia reduced viral replication to the same extent as stimulation with recombinant type I IFN, whereas STING-deficient microglia failed to transfer antiviral activity to neurons (Fig. 6b). Although the neurons did not produce type I IFN (Fig. 3c,d), they did respond to type I IFN treatment with a classical ISG expression profile irrespetive of their STING genotype (Fig. 6c–g; Supplementary Fig. 8), and this response was also evoked by supernatants from HSV-1-infected WT microglia (Fig. 6g). Supernatants from HSV-1-infected microglia also evoked anti-HSV-1 activity in astrocytes, but unlike what was observed in the neurons, this occured in a manner independent of STING (Fig. 6i). Interestingly, the STING-independent nature of the antiviral signals delivered from HSV-infected microglia to astrocytes is in line with the findings *in vivo*, where viral infection in astrocytes was not affected by STING deficiency (Fig. 2d,e).

To describe how microglia may employ the cGAS–STING pathway to prime type I IFN production in astrocytes, we first isolated supernatants from microglia stimulated with synthetic dsDNA, and added the conditioned media to astrocytes, which were subsequently stimulated with the dsRNA analogue poly(I:C). We found that media from DNA-treated microglia significantly potentiated IFNβ stimulation by poly(I:C) (Fig. 6j). Addition of poly(I:C) directly to the medium stimulated TLR3-dependent type I IFN responses in astrocytes (Supplementary Fig. 9), and we previously reported that this cell type relies on TLR3 to sense HSV-2 infection^11^. Therefore, we examined if HSV-1-induced expression of IFNβ and the ISG CXCL10 was affected by TLR3 deficiency. As seen in Fig. 6k,l, TLR3 was essential for full HSV-1-induced expression of IFNβ and CXCL10 by both resting and type I IFN-primed astrocytes. Finally, we examined whether the activation of the cGAS–STING pathway in microglia could transfer priming signals to astrocytes. Importantly, astrocytes cultured with conditioned medium from dsDNA-stimulated WT but not STING-deficient microglia responded to HSV-1 infection with strong induction of IFNβ mRNA expression (Fig. 6m). The priming of virus-induced IFNβ expression in astrocytes by microglia activated through the cGAS–STING pathway correlated with the expression of TLR3, which was induced by type I IFN treatment and medium from HSV-1-infected or dsDNA-stimulated WT microglia (Fig. 6n). Together with the data presented in Fig. 3c, these data demonstrate that resting astrocytes rely on the STING pathway to produce type I IFN in response to HSV-1 infection, and that microglia on DNA sensing can prime the TLR3 pathway in astrocytes to enable this cell type to respond to HSV-1 infection with high expression of type I IFNs.

Collectively, the data demonstrate that HSV-1-infected microglia transfer STING-dependent signals to adjacent cells, leading to the antiviral activity in neurons and priming of the TLR3 pathway in astrocytes.

## Discussion

A large proportion of the human population is infected with HSV-1, but only a small minority experience HSE^23^. In recent years, it has emerged that the innate immune system, including the TLR3-type I IFN axis is important for protection against HSV-1 infection in the CNS and prevention of HSE^6^,^7^,^8^,^9^,^10^. In this work, we demonstrate that a defective cGAS–STING pathway renders mice highly susceptible to HSE-related disease after infection at an epithelial surface in the periphery. Moreover, we show that among CNS cells, particularly microglia utilize the cGAS–STING pathway to orchestrate the antiviral response through at least two mechanisms acting in a paracrine manner: antiviral action of type I IFN, and priming of innate immune sensing pathways, including the TLR3 pathway (Supplementary Fig. 10).

Previous studies in murine models have suggested that TLR2 deficiency protects against the pathological inflammatory response in the brain after intracranial HSV-1 infection^24^, whereas lack of TLR9 did not have an impact on the viral replication in the brain after ocular or intracranial infection^25^,^26^. Moreover, TIR-domain-containing adapter-inducing interferon-β (TRIF)-deficient mice exhibited markedly increased susceptibility to HSE-related disease and impaired viral control in the brain after intranasal HSV-1 infection, whereas mitochondrial antiviral-signaling protein (MAVS)-deficient mice had a modest but significant phenotype in the same model^27^. It has been reported that cGAS and STING-deficient mice exhibit impaired defense against HSV-1 in the CNS after infection through the intravenous route^22^,^28^. In this model, the entry of the virus into the CNS is not well understood, and includes the hematogenous route^29^. By contrast, the natural route of entry for HSV-1 into the CNS is via sensory neurons. Thus, together with the previous report on HSV-1 infection in the CNS after intranasal infection of TRIF-deficient mice^27^, and the extensive literature on defects in the TLR3 pathways in HSE patients^6^,^7^,^8^,^9^,^10^, the present study corroborates the cGAS–STING and the TLR3–TRIF pathways as the main innate sensing pathways conferring control of HSV-1 infection in the CNS. Although we observed very similar phenotypes for cGAS- and STING-deficient mice, it should be mentioned that we cannot formally exclude that STING also has cGAS-independent functions in defense against HSV-1 in the CNS^30^,^31^.

Parallel to our work, David Leib and colleagues studied HSV-1 infection in STING-deficient mice^20^. While Parker *et al*. used the KOS and 17 strains for their ocular infection experiments, we mainly used the McKrae strain, since it is more neuro-invasive. Some of our results with the McKrae strain were repeated with the KOS strain. We found that more STING-deficient than WT mice succumbed after ocular infection with the McKrae strain, and Parker *et al*. found the same for the 17 strain^20^. Both studies show increased viral load in the eyes and trigeminal ganglia of STING-deficient mice after ocular infection with the KOS strain, and also that the KOS strain does not replicate to high titres in the brain even in the absence of STING^20^. To overcome this, Parker *et al*. injected virus directly into the brain and found lower levels of IFNβ in the brains of infected Sting^−/−^ mice, in agreement with our data. However, interestingly, expression of inflammatory cytokines was elevated in corneas of STING-deficient mice^20^. This may be induced by other PRRs such as TLRs, which have been reported to be involved in sensing of HSV-1 in the cornea^32^. Therefore, these two studies together establish an essential role of STING in control of HSV-1 after corneal infection, and our choice of the McKrae strain has allowed us focus more on the infection in the CNS.

A central finding of the present study is that microglia represent the main source of type I IFN during HSV-1 infection and utilizes the cGAS–STING pathway to induce this response. Moreover, the cGAS–STING pathway is important for cell-autonomous control of the viral replication in microglia but not in astrocytes and neurons. This indicates that in the CNS, the cGAS–STING pathway is important mainly for the antiviral action of microglia, which in turn disseminate antiviral activities to other cell types of the CNS. The importance of microglia in HSV-1-induced type I IFN production is consistent with work by others^17^. In this work, we identified two mechanisms through which HSV-infected microglia can transfer antiviral activities to other cell types in a STING-dependent manner. First, microglia-derived type I IFN leads to significant inhibition of HSV-1 replication in the highly permissive neurons. The ability of type I IFN to inhibit HSV replication in neurons is in agreement with earlier work by us and others^11^,^33^,^34^. Previous work has demonstrated lower steady state and type I IFN-induced levels of several ISGs associated with cell death in neurons as compared with mitotic cells^33^. Future work should resolve whether type I IFN activates a specialized antiviral program in neurons. Second, we found that type I IFN and supernatants from HSV-infected microglia upregulated TLR3 expression in astrocytes, and this cell type accordingly did acquire elevated responsiveness through the respective pathways. Given the well-described role of the TLR3–TRIF pathway in control of HSV-1 in the CNS in humans and mice^6^,^7^,^27^, these data suggest that a defective cGAS–STING pathway also indirectly hampers the ability to evoke full antiviral activity through the TLR3–TRIF pathway. The concept of cross-talk between the type I IFN-inducing pathways in the CNS during HSV-1 infection is further supported by the previous finding that TRIF-deficient mice, along with higher viral load, have significantly elevated IFN-β expression in the brain^27^. On the basis of the data in the present work, we suggest that the elevated type I IFN-β expression in TRIF-deficient mice is stimulated through the cGAS–STING pathway. It is tempting to speculate that the detection of HSV-1 through multiple type I IFN-inducing pathways that interact in a manner involving multiple cell types enables the organism to achieve optimal antiviral activity, with the lowest possible amounts of type I IFN. Such a scenario would render the host antiviral response less sensitive to viral evasion and targeting of specific cell types or antiviral pathways.

Collectively, this work demonstrates that the STING-dependent DNA-sensing system represents a key pathway to control HSV-1 replication in the CNS, and that this is highly dependent on viral DNA recognition in microglia. Thus, by possession of strong responsiveness through the cGAS–STING pathway, microglia act upstream in an intercellular network in the CNS during HSV-1 infection. Through this network, microglia disseminate antiviral activities through the tissue and also primes select innate pathways in other cell types in the CNS, which together orchestrate the antiviral response.

## Methods

### Mice

C57BL/6, STING^gt/gt^ and cGAS^−/−^ mice were bred at Taconic M&B. Isoflourane (Abbott), or (Ketamine (MSD Animal Health) and Xylazin (RompunVet)) was used to anaesthetize mice. All described animal experiments have been reviewed and approved by Danish government authorities and hence comply with Danish laws (The Animal Experiments Inspectorate, Slotsholmsgade 10, 1216 København K, Denmark). All efforts were made to minimize suffering and mice were monitored daily during infection. The mice were not randomized but after HSV infection, the information about mice strain and treatment were blinded to the investigators. No animals were excluded from the analysis.

### Viruses and reagents

Dulbecco's Modified Eagle Medium (DMÈM) was obtained from BioWhittaker and supplemented with 100 IU ml^−1^ penicillin, 100 μg ml^−1^ streptomycin and LPS-free FCS (BioWhittaker). IFN-β/α (PBL), and ProLong Gold, DAPI, TRIzol, Poly I:C (all from Invitrogen) Lipofectamine 2000 (Invitrogen) were used in the experiments described below. HSV-1 (strains KOS and McKrae) was grown in Vero cells. The Vero cells used were from the lab stock. The titres of the stocks used were 8–14 × 10^9^ PFU ml^−1^. Titres were determined by plaque assay on Vero cells. Beriglobin was used to neutralize extracellular HSV-1 (CSL Behring) and fully neutralized the virus in the dilutions used to calculate titres. Both strains were used for infection of mice, while only KOS strain was used for *in vitro* stimulation. GFP-HSV-1 (HSV-1-expressing eGFP driven by the CMV promoter) and HSV-1 McKrae were kind gifts from David Leib, while HSV-1 (KOS) was a gift from Peter O'Hare.

### Murine ocular HSV-1 infection model

Age matched (6–12-week old) male mice, were anaesthetized with intraperitoneal (i.p.) injection of ketamine (100 mg kg^−1^ body weight) and xylazine (10 mg kg^−1^ body weight). Corneas were scarified in a 10 × 10 crosshatch pattern and mice were either inoculated with 1 × 10^6^ PFU HSV-1 in 5 μl of infection medium (DMEM containing 200 IU ml^−1^ penicillin and 200 μg ml^−1^ streptomycin), or mock infected with 5 μl of infection medium. Mice were scored for disease and weighed at the indicated times post infection. The scoring was performed as blinded study, largely following previous descriptions by others^20^ with the following minor modifications: hair loss (0: none, 1: minimal periocular hair loss, 2: moderate periocular hair loss, 3: severe hair loss limited to periocular, 4: hair loss severe and extensive); hydrocephalus (0: none, 1: minor bump, 2: moderate bump, 3: large bump); symptoms related to neurological disease (0: normal, 1: jumpy, 2: uncoordinated, 3: hunched/lethargic, 4: unresponsive/no movement); eye swell/lesions (0: none, 1: minor swelling, 2: moderate swelling, 3: severe swelling and skin lesions, 4: lesions extensive). Mice were killed at the specified times post infection or once they met end point criteria. Eye wash was collected by gently proptosing each eye and wiping a sterile cotton swab three times around the eye in a circular motion and twice across the center of the cornea. The swabs were placed in 0.5 ml of DMEM medium and stored at −80 °C until the titre was determined.

Brains, brain stems and trigeminal ganglia were frozen immediately at −80 °C. Tissues were homogenized with steel beads (Qiagen) in Tissuelyser (II) (Qiagen) for 3 min at frequency (30 s). One half was used for RNA isolation with TRIzol and the other half was used for virus plaque assay as described^11^.

### Preparation and culture of organotypic brain slices

Organotypic brain slice cultures were prepared and grown by the interface culture method as also previously described^35^,^36^. In brief, postnatal day 0–1 mice were quickly decapitated and the brains isolated under aseptic conditions. After removal of the meninges and isolation of the brain stem (midbrain and pons, excluding medulla oblongata), tissue blocks were sectioned transversely at 400 μm using a McIlwain tissue chopper (Mickle Laboratory). The slices were immediately transferred to chilled Gey's balanced salt solution (Gibco) supplemented with 30 mmol l^−1^ glucose and 30 mmol l^−1^ KCl (Merck) for subsequent separation and trimming of the slices. Three tissue slices from the same brain were placed on each semiporous membrane insert (pore size 0.4 μm; Millipore, Bedford, MA, USA) and transferred to six-well culture trays with 1 ml serum-containing culture medium per well. The medium was composed of 25% heat-inactivated horse serum (Gibco), 25% Hanks balanced salt solution (Gibco), 25 mmol l^−1^ D-glucose (Merck) in OPTI-MEM (Gibco). The organotypic brain slice cultures were grown at 36 °C in 5% CO_2_ for 48 h before HSV-1 infection (Mckrae; 5 × 10^3 ^PFU in 3,5μl per brain slice). Two days post infection culture medium and six days post infection, brain slices were collected, homogenized and the viral loads determined as described above.

### Isolation and culturing of primary cells

Neurons were isolated from P 0–3 mice using the Neural Tissue Dissociation Kit (MACS, Miltenyi Biotec) according to the manufacturer's instructions. The cells were pre-plated for 45 min in culture flasks to avoid the quickly attaching non-neuronal cells. The floating neurons were seeded on poly-D-lysine (Sigma-Aldrich) and laminin (Invitrogen) pre-coated 24-well plates. The cells were cultured in Neurobasal-A medium (Gibco) containing B-27 Supplement (Gibco), 2 mM GlutaMAX (Gibco), 100 μg ml^−1^ Primocin (Invivogen) and 20 μM floxuridine+20 μM uridine (Sigma), and media was changed every second day. On day 7, purity of the cells was checked by flow cytometry and the neurons were infected with indicated MOI of HSV-1 and washed 1 h following infection. For RNA analysis, the few microglia present in the culture was removed by fluorescence-activated cell sorting (FACS) 500 CD45^−^ cells in to each well on a 96-well plate and analysed on BioMarker (Fluidigm) or by regular quantitative PCR (qPCR).

The astrocytes and the mixed glial cells were isolated and cultured as previously described^11^. In brief, for isolation of glial cells from mice, neonatal cerebra were collected, trypsinized for 25 min and filtered through a 70-μm pore size filter. Cells of one cerebrum were then seeded on 1 uncoated 75 cm^2^ culture flask and incubated with 40 ml DMEM containing 10% FCS. The medium was replenished on days 1 and 4 after plating. Henceforth, either microglia or astrocytes were isolated:

Astrocytes were isolated using the following method: glial cells were passaged two to three times and shaken at 250 r.p.m. for 48 h, supernatant was aspirated and the remaining adherent cells were predominantly astrocytes.

The microglia was isolated as described elsewhere^37^. Shortly, the confluent mixed glial cultures were grown in DMEM-F12 (Lonza) with FBS for 3–4 weeks, and the astrocyte layer growing above the microglia were removed using mild trypsinization (0.05–0.12%) in the presence of 0.2–0.5 mM EDTA and 0.5–0.8 mM Ca^2+^. The remaining adherent microglia cells were isolated by trypsinization and seeded for the assay.

The purity of the primary cells was in the range 89–97% depending on the cell type (Supplementary Fig. 4). When using supernatants from microglia cultures to treat other cell types, the supernatants were first ultraviolet-inactivated for 6 min.

### Immunohistochemistry

Immunohistochemistry was performed as described previously^11^. Briefly, the mice were perfused with 4% formaldehyde fixative through the circulatory system, and postfixed with phosphate-buffered 4% formaldehyde and brain sections were cut at 60-μm thickness and every sixth section was stained. The antibody retrieval was performed at 80 °C for 20 min with Target Retrieval Solution (Dako). The sections were treated with methanolic H_2_O_2_ (3% H_2_O_2_ and 1% absolute methanol) for 30 min to inactivate endogenous peroxidase activity. The sections were then blocked with 0.5% bovine serum albumin (BSA) in TBS for 1 h at room temperature (RT), and incubated overnight at 4 °C with rabbit polyclonal anti-HSV-1 (1:500; DakoCytomation, B0116) antibody in tris-buffered saline (TBS) containing 0.1% BSA, 0.3% Triton-X, followed by several washes in TBS containing 0.3% Triton-X TBS and staining with and the secondary antibody, donkey anti-rabbit HRP (1:500; Sigma-Aldrich) for 1 h at RT. Horseradish peroxidase-labelled antibodies were developed with chromagen diaminobenzidine. Finally, the slides were rinsed in water, dehydrated and prepared for imaging. The imaging was performed on Zeiss AX10, Scope A1 microscope using × 20 and × 2.5 objectives.

### Confocal microscopy of tissue sections and glia cell cultures

For astrocyte, microglia, neuronal and viperin staining, 50-μm cryosections (HM 500 OM, Mikrom) were stained and imaged as described above and previously^11^. The sections were incubated in TEG buffer (10 mM Tris Base, 0.5 mM EGTA, pH 9) at 60 °C overnight and subsequently treated with methanolic H_2_O_2_ (3% H_2_O_2_ and 1% absolute methanol) for 30 min. The sections were washed and incubated with primary antibodies at 4 °C overnight, including mouse monoclonal anti-NeuN (1:300; clone A60, Millipore), mouse monoclonal anti-GFAP (1:400; clone G-A-5, Sigma-Aldrich), mouse monoclonal anti-S100 (1:200; Clone 4C4.9, Abcam), mouse monoclonal anti-viperin (1:400; clone MaP.VIP, Milipore), rabbit polyclonal anti-HSV-1 (1:500; DakoCytomation, B0116) and goat polyclonal anti-Iba1 (1:400; Abcam; ab5076). As a control for staining, we used secondary antibody alone and isotype control in case the primary antibody was monoclonal. After several washes in TBS containing 0.3% Triton-X TBS, the secondary antibodies (all at dilution 1:500; Invitrogen; chicken anti-mouse Alexa Fluor 647, donkey anti-rabbit Alexa Fluor 568, chicken anti-goat Alexa Fluor 488) were incubated for 1 h at 20 °C. Nuclei were stained with 4,6-diamidino-2-phenylindole for 6 min. The sections were washed five times in 0.3% Triton-X in TBS and mounted using ProLong Gold. Sections were imaged on a Zeiss LSM 710 laser scanning microscope. Zen 2010 acquisition software and ImageJ (http://rsbweb.nih.gov/ij/) were used for imaging and analysis. For microscopy on cells cultured *in vitro,* isolated microglia and astrocytes were mixed 1:1 and grown together on glass cover slips for 48 h and infected with HSV-1 (KOS, MOI 10) for 4 h. The cells were fixed and stained as described^11^. The antibodies used were mouse anti-GFAP (1:400; Sigma-Aldrich; GA5), Rabbit anti-STING (1:100; IMGENEX, IMG-6485A); and secondary antibodies (1:300), Alexa Fluor 488 Donkey anti-mouse IgG (A21202) and Alexa Fluor 568 Donkey anti-rabbit IgG(A10042) from Life Technologies.

### RNA isolation and quantitative RT–PCR

The RNA purification and qPCR was preformed as described^11^, with the following primers from Applied Biosystems: TLR3 (Mm01207404_m1), cGAS (Mm01147497_m1), STING (Mm01158117_m1), MX1 (Mm00487796_m1), ISG15 (Mm01705338_s1), Viperin (Mm00491265_m1), IL-6 (Mm00446190_m1), IFN-β (Mm00439552_s1), CXCL10 (Mm00445235_m1), HSV-1 gB (the forward primer (5′-CGC ATC AAG ACC ACC TCC TC-3′), the reverse primer (5′-AGC TTG CGG GCC TCG TT-3′) and probe: 5′-CGG CCC AAC ATA TCG TTG ACA TGG C-3′), Iba-1 (Mm00479862_g1) and HSV-1 LAT (The forward primer (5′-ACCCACGTACTCCAAGAAGGC-3′), the reverse primer (5′-TAAGACCCAAGCATAGAGAGCCA-3′ and probe (5′-TCCCACCCCGCCTGTGTTTTTGT-3′)). Levels of mRNAs of interest were normalized to β-actin using the formula 2^Ct(βactin)−Ct(mRNA X)^. The resulting normalized ratio were either presented directly in the figures or further normalized to the normalized ratio of untreated WT cells as specified in the figure legends.

### Fluidigm

Complementary DNA (cDNA) was isolated from 500 FACS-sorted cells and specific targets were pre-amplified in one step by using the CellDirect One-Step qRT-PCR kit (Invitrogen) according to the manufacturer's instructions. Briefly, 500 FACS-sorted cells were combined with SUPERase-In (Ambion), SuperScript II RT/Platinum Taq mix (Invitrogen) and 0.2 × of all primers used in the present study (TaqMan gene expression assays, Applied Biosystems). The PCR with reverse transcription (RT–PCR) was performed using the thermal cycling protocol: RT (incubated 50 °C for 15 min),Taq activation (95 °C for 2 min), followed by 15 cycles of (95 °C at 15 s and 60 °C at 4 min). Pre-amplified cDNA was diluted at least 1:5 in low EDTA TE-buffer (VWR—Bie & Berntsen) before real-time qPCR on the BioMarker (Fluidigm). qPCR was performed in the 48.48 Dynamic Array Integrated Fluidic Circuits (Fluidigm) combining 48 pre-amplified samples with some of the primers as mentioned above. The following reagents were used for 48 reactions of pre-sample mix: 3 μl of TaqMan gene expression master mix (Applied Biosystems), 0.3 μl of 20 × GE sample loading reagent (Fluidigm), 6 μl low EDTA TE buffer (VWR—Bie & Berntsen) and 2.7 μl diluted pre amplified cDNA. Primer mixes were prepared using 2.5 μl of 20 × TaqMan gene expression assays (Applied Biosystems) as described above and 2.5 μl of 2 × assay loading reagent (Fluidigm). The 48.48 Dynamic Array was primed in the IFC controller (Fluidigm) before loading of sample and primer. Sample mix, including cDNA (5 μl) and primer mix (5 μl) was dispensed into appropriate inlets and loaded into the chip in the IFC controller, combining each sample with each primer pair in separate reactions. The plate was placed in the BioMark PCR instrument (Fluidigm) and the standard protocol was followed. Data was acquired using the Fluidigm Real-Time PCR Analysis software 3.0.2 (Fluidigm).

### Enzyme-linked immunosorbent assay and type I IFN bioassay

Supernatants from primary CNS cells were inactivated by irradiation with ultraviolet light for measurement of type I IFN bioassay or CXCL10 by enzyme-linked immunosorbent assay as described previously^38^.

### Western blotting

Eye and brain tissues were lysed and used for western blotting as described previously^38^. Briefly, the tissues were lysed in RIPA buffer in the presence of protease and phosphatase inhibitors then subjected to homogenization and centrifugation. The cleared supernatants were sonicated, and samples were subjected to SDS–polyacrylamide gel electrophoresis and immunoblotting. The blots were blocked with 2.5% BSA and probed with the following primary antibodies: polyclonal sheep anti-STING (1:100; R&D, AF-6516) and mouse monoclonal anti-Vinculin (1:1,000; Sigma, clone hVIN-1,V9131). Appropriate peroxidase-conjugated secondary antibodies were used for development (Jackson ImmunoResearch). Secondary antibodies were as follows: anti-goat, catalogue no. 705-306-147; anti-rabbit, catalogue no. 711-035-152; and anti-mouse, catalogue no. 715-036-150 (all used at dilution 1:10,000).

### FACS sorting and flow cytometry

The stimulated neurons were stained for CD45, and 500 CD45^−^ cells were FACS sorted into a 96-well plate and used for mRNA quantification either by Fluidigm or by regular qPCR as described above. In the assays with GFP-HSV-1-infected neurons, the CD45^−^ cells were further divided into GFP^−^ and GFP^+^ populations before RNA analyses. Similarly, in GFP-HSV-1-infected mixed culture of astrocytes and microglia, both CD45^+^ and CD45^−^ cells were divided in to GFP^+^ and GFP^−^ population of 500 cells. For CD45 staining, cells were first blocked for 15 min with mouse IgG (Jackson ImmunoResearch Inc.) and purified rat anti-mouse CD16/CD32 (Mouse BD Fc Block) to prevent non-specific binding. The antibodies rat anti-CD45-APC (Clone 30-F11,BD, Pharmingen) and APC Rat IgG2b, κ isotype control (BD, Pharmingen) were added and incubated in (1:200) for 15 min at 20 °C in the dark. After washing with PBS/BSA (PBS, pH 7.4, supplemented with 0.5% BSA) the cells were analysed or sorted on a FC500 flow cytometer (Beckman Coulter) or a FACSAria III (BD Biosciences). Subsequently, FlowJo software version 8.8.3 (Tree Star Inc.) was used for data analysis. For quantifying primary cells, the cells were trypsinized, fixed in methanol, permeabilized in 0.5% Triton X-100, and the blocking and antibody staining and analysis were done as described above for CD45 staining. The antibodies used were mouse anti-GFAP-PE (1:200; Clone GA5, Millipore) and isotype control IgG1K-PE (Millipore). The staining for neurons was done similarly, where blocking was done in 10% BSA and the antibody used was mouse monoclonal anti-NeuN (1:300; clone A60, Millipore), together with the secondary antibody chicken anti-mouse Alexa 647 (1:500), or the secondary antibody alone. For quantifying microglia from adult mouse brains, the brain stems were made into single-cell suspension by carefully triturating with Pasteur glass pipettes (VWR) with decreasing diameter until it became a homogenous cell suspension. Large debris and cell clusters were removed by filtration through a 70-μm cell strainer (BD Biosciences #352350) and myelin debris was reduced by three-density step gradient of Percol (Sigma) as described^39^. The cells were then washed and stained as described above with anti-CD45-APC and rat monoclonalanti-CD11b-BV421 1:300 (clone M1/70, Biolegend) and their isotype controls (APC Rat IgG2b, κ) and (BV421 Rat IgG2b, κ). The microglia were also stained with mouse monoclonal anti-Iba-1-FITC (1:100; Clone 1022-5, Abcam) or isotype control mouse IgG2b–FITC(Abcam). The gating strategies used for the flow cytometry experiments are shown in Supplementary fig. 11.

### Statistics

For statistical analysis of data on cytokines, chemokines and virus titres, we used two-tailed Student's *t*-test when the data exhibited normal distribution, and Wilcoxon rank-sum test when the data set did not pass the normal distribution test. *P*≤0.05 were considered to reflect statistically significant differences between compared groups. Symbols for *P*-values used in the figures: *0.01<*P*<0.05, **0.001<*P*<0.01, ***P<0.001. The data shown are representative of one out of three or more experiments and all samples in a group are biological replicates.

### Data availability

The data that support the findings of this study are available from the corresponding author upon reasonable request.

## Additional information

**How to cite this article:** Reinert, L. S. *et al*. Sensing of HSV-1 by the cGAS–STING pathway in microglia orchestrates antiviral defense in the CNS. *Nat. Commun.* **7,** 13348 doi: 10.1038/ncomms13348 (2016).

**Publisher's note:** Springer Nature remains neutral with regard to jurisdictional claims in published maps and institutional affiliations.

## Supplementary Material

Supplementary Information Supplementary Figures 1-11
