# Supplementary material for: Sensing of HSV-1 by the cGAS–STING pathway in microglia orchestrates antiviral defence in the CNS
Source: Nat Commun. 2016 Nov 10;7:13348. doi: 10.1038/ncomms13348 (PMC5109551; doi:10.1038/ncomms13348)
Supplement: Supplementary Information — Supplementary Figures 1-11 [file ncomms13348-s1.pdf]

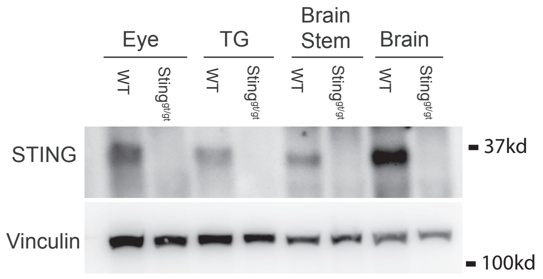

Supplementary Figure 1. Expression of STING in WT and Sting$^{gt/gt}$ mice after ocular infection with HSV-1 (McKrae). Homogenates from eyes, trigeminal ganglia (TG), brain stem and brain were subjected to SDS-PAGE and western blotting and probed with antibodies against STING and Vinculin.

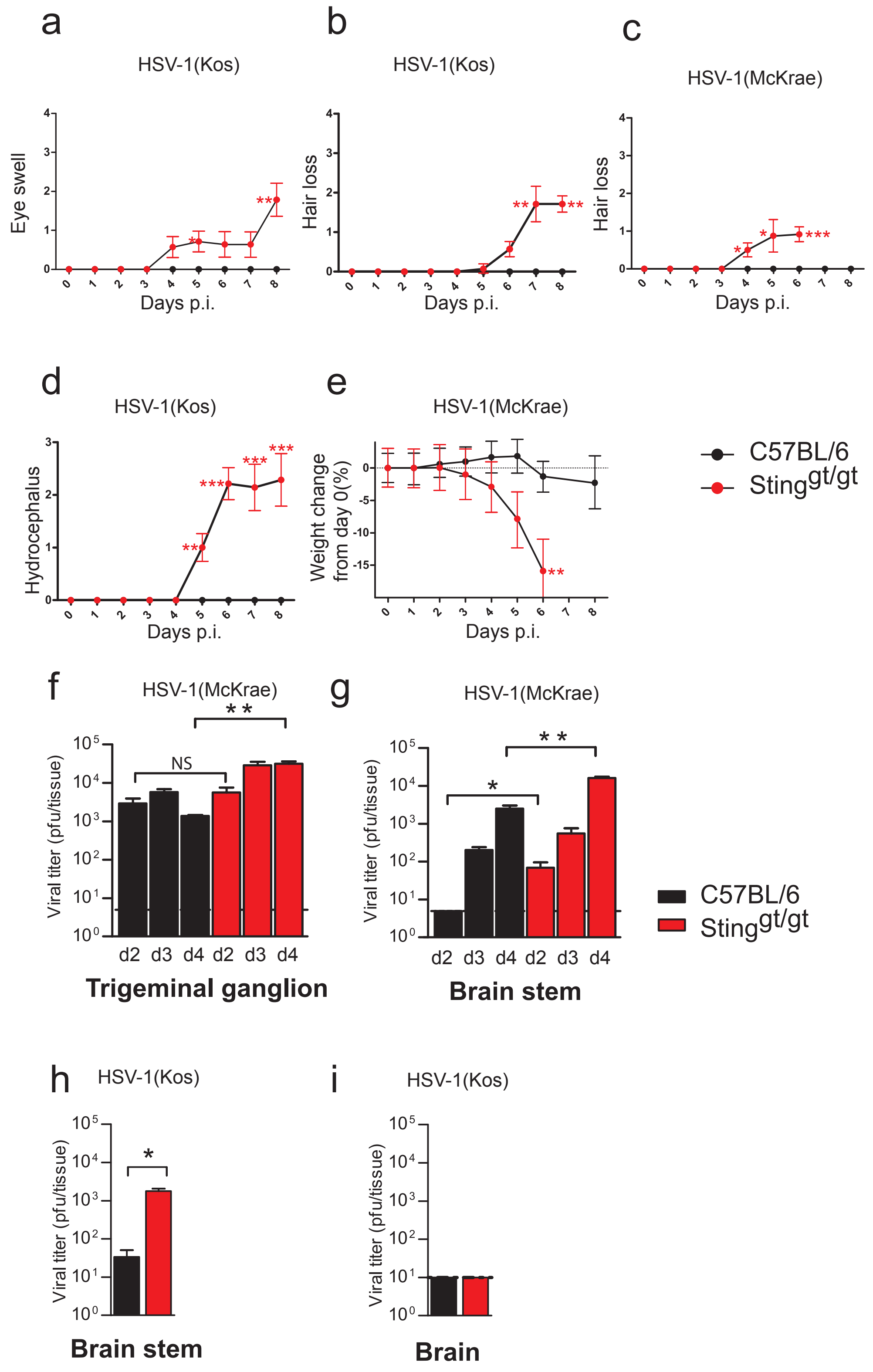

Supplementary Figure 2. Clinical and virological parameters in WT and Sting$^{gt/gt}$ mice infected with HSV-1 strains. (**a-e**) WT and Sting$^{gt/gt}$ mice were infected with 1x10^{6} PFU/eye of HSV-1 (strains KOS or Mckrae), and followed over time for scoring of different clinical parameters, and measurement of weight. (**f-i**) Homogenates of the indicated tissue isolated from WT and Sting$^{gt/gt}$ mice infected for (**f-g)** 2, 3 or 4 days with McKrae or (**h-i**) for 6 days with HSV-1 KOS were analyzed for viral load by plaque assay. All data in this figure are shown as mean values +/- SEM. n= 6-9 per group. *, 0.01<p<0.05; **, 0.001<p<0.01; *** p<0.001; NS, not significant. In figure (**a-e)** The red asterisks indicate p-values between WT and KO at specific days p.i. In figure (**f-g,i**) the dashed line indicates the detection limit.

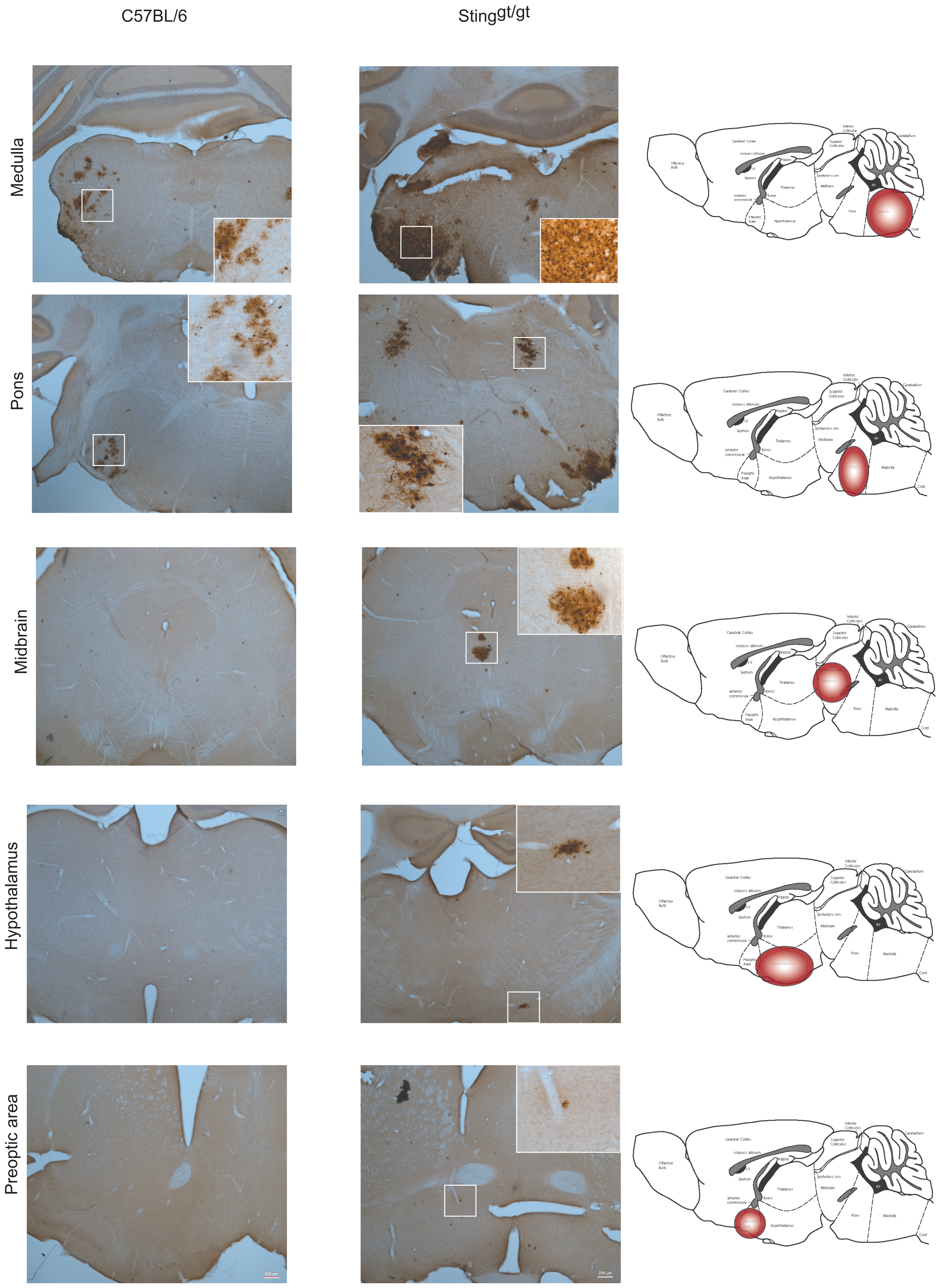

Supplementary Figure 3. Whole-brain sectioning of brains from perfused mice infected with HSV-1. Whole brains from 6 WT and 6 Sting$^{gt/gt}$ mice infected for 6 days with HSV-1 were sectioned in 60 μm sections and every sixth section was stained with an antibody against HSV-1. The areas highlighted with red in the illustrations of the mouse brains to the right, indicate the brain area where the sections shown to the left originate. Images are shown from medulla, pons, midbrain, hypothalamus, and preoptic area. The images of sections of medulla from HSV-1-infected WT and Sting$^{gt/gt}$ mice are identical to the images shown in Figure 1j. The original magnifications are 2.5x and 20x for the zoomed in images.

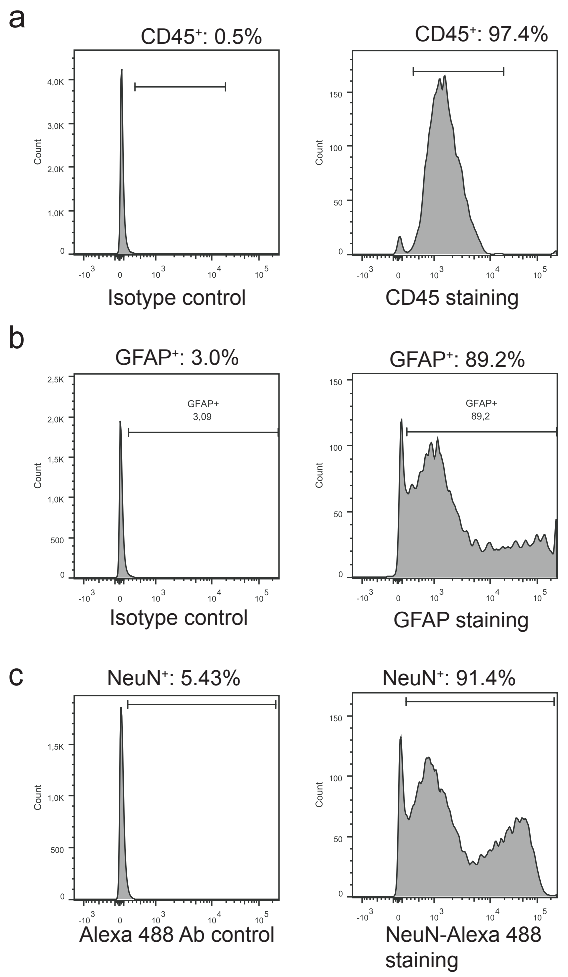

Supplementary Figure 4. Microglia, astrocytes, and neurons were isolated, and differentiated from brains of new-born mice as described in the methods section. Prior to each set of experiments, the cell populations obtained were examined for expression of (**a**) CD45, microglia; (**b**) GFAP, astrocytes; (**c**) NeuN, neurons.

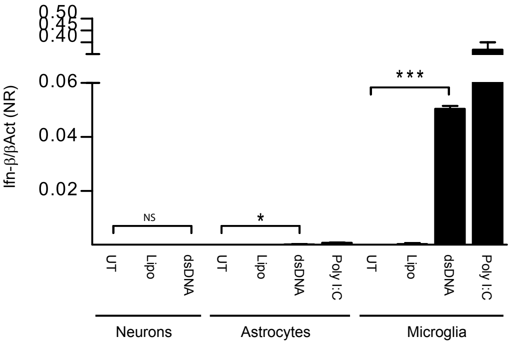

Supplementary Figure 5. DNA-stimulated production of type I IFN in microglia, astrocytes and neurons. WT cultures treated for 6 h with Lipofectamine, dsDNA (2 μg/ml) or poly(I:C) (5μg/ml). Total RNA from astrocytes neurons or microglia cultures was analyzed for expression of IFN-b by RT-qPCR. Data are presented as mean (normalized to β-actin levels) +/- SEM, n=5-6 per group.

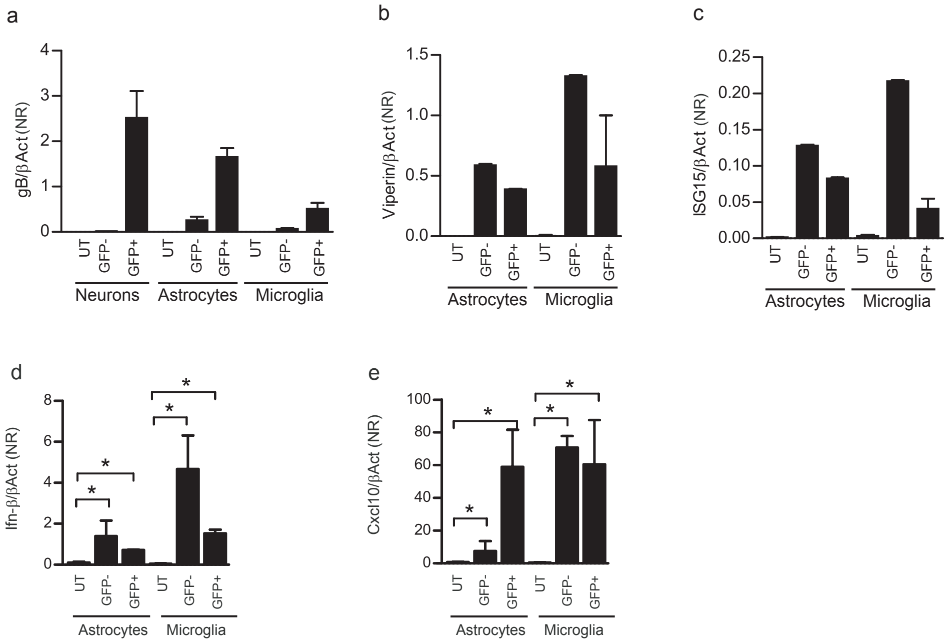

Supplementary Figure 6. Expression of viral genes and IFN-stimulated genes in cultures of CNS cells infected with HSV-1. (**a**) Cultures of neurons, astrocytes, and microglia were infected with HSV-1 expressing eGFP from the CMV promoter (MOI 3). 6 h later, the cells were sorted into GFP+ and GFP- positive populations. Total RNA was analyzed together with uninfected controls for expression of gB mRNA. The data are presented as means normalized to β-actin levels +/- SEM. (**b- e**) Mixed cultures of astrocytes and microglia were infected with eGFP HSV-1 (MOI 3, **b-c**) or (MOI 1, **d-e**). Six h later, the cells were sorted into GFP^{+} and GFP^{-} populations, and further sorted into astrocytes and microglia. Total RNA from the 4 populations was analyzed together with uninfected controls for expression of Viperin, Isg15, Ifn-β, or Cxcl10. The UT refers to the GFP^{-} population from uninfected cell cultures. The data are presented as means to β-actin levels +/- SEM. *, $0.01<p<0.05$, n=5-6 per group.

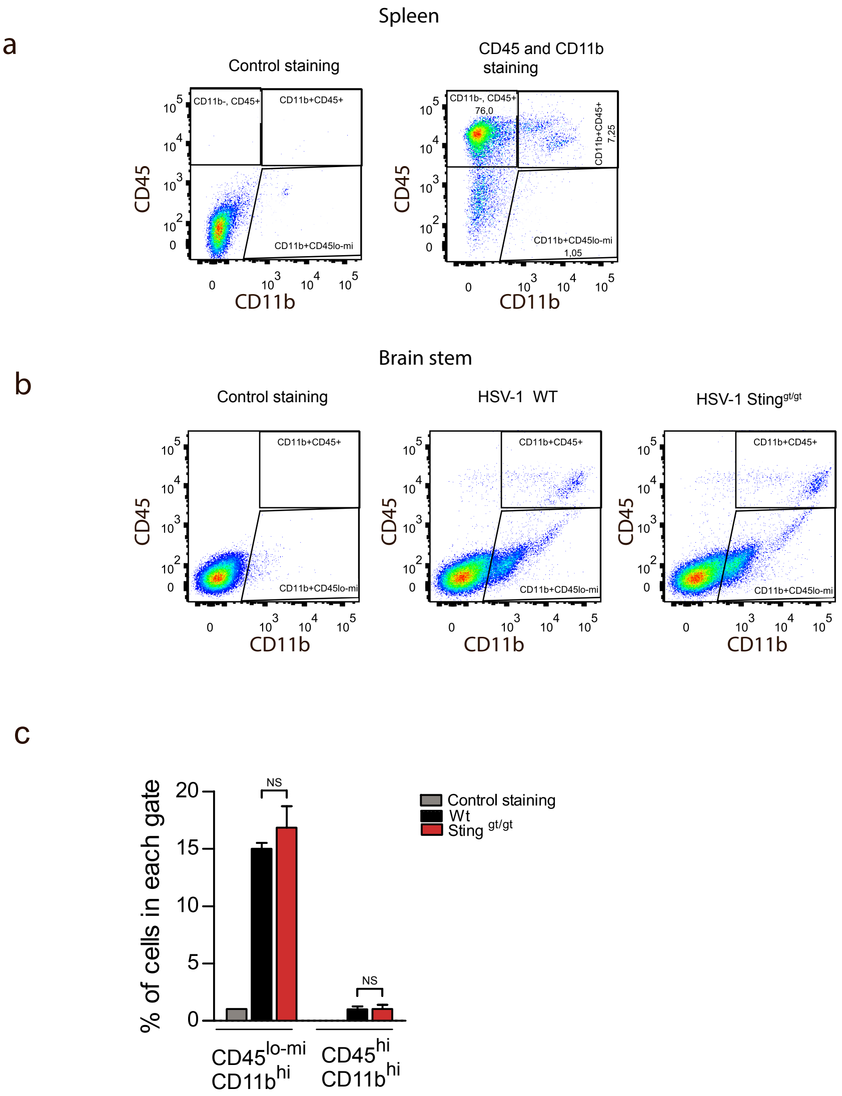

Supplementary Figure 7. Similar levels of microglia are present in the infected area of the brains of WT and STING$^{gt/gt}$ mice. (**a**) To distinguish between CD45^{-}, CD45$^{lo\text{-}medium}$, and CD45^{hi} cells, splenocytes from an uninfected C57BL/6 mouse were stained with anti-CD45. The isotype control staining, were used for setting the gates used for the infected brain stem. n=3 for splenocytes (**b and c**) Brain stems were isolated from STING$^{gt/gt}$ and WT mice at day 6 p.i. Single cell suspension were cleared of myelin debris, and stained for CD45 and CD11b. The gating and percentage of cells in each gate, microglia (CD45$^{lo\text{-}medium}$ and CD11b^{hi}) and bone marrow-derived macrophages (CD45^{hi} and CD11b^{hi}) are quantified and presented as means +/- SEM (n=3-6).

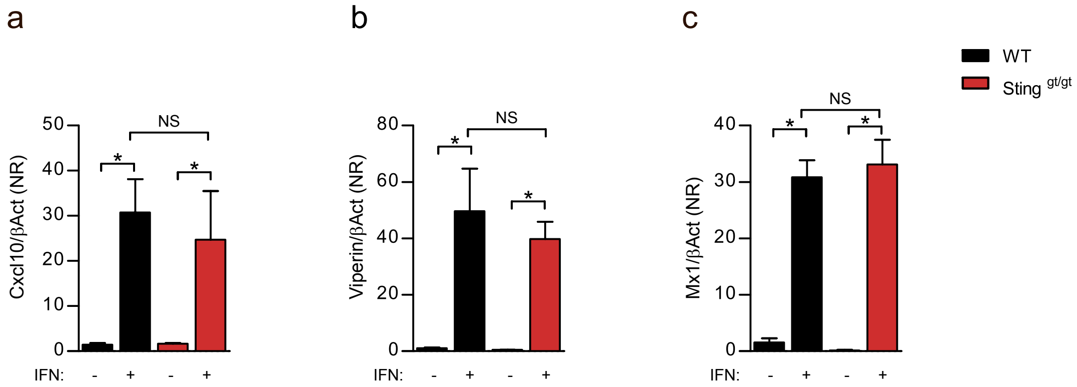

Supplementary Figure 8. Type I IFN induces ISG responses in neurons independent of STING (**a, c**). Total RNA from neuron cultures stimulated with IFN-α/β (25 U/ml) or medium alone for 17 h was analyzed for expression of the ISGs Cxcl10, Viperin, or Mx1. Data are presented as means +/- SEM. All RT-QPCR data in this figure were normalized to β-actin levels and are presented as (means +/- SEM) fold induction relative to the WT UT. Symbols for p-values used in the figures: *, 0.01<p<0.05; **, 0.001<p<0.01; *** p<0.001; NS, not significant, n=5-8 per group.

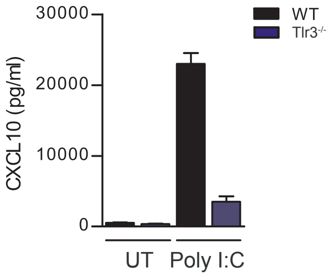

Supplementary Figure 9. Stimulation with extracellular poly(I:C) evokes TLR3-dependent responses in astrocytes. WT and Tlr3$^{-/-}$ astrocyte cultures were treated with poly(I:C) added directly to the medium (5 μg/ml). Supernatants were harvested 24 h post treatment, and levels of CXCL10 protein were determined by ELISA. Data are presented as means +/- SEM, n=5-6 per group.

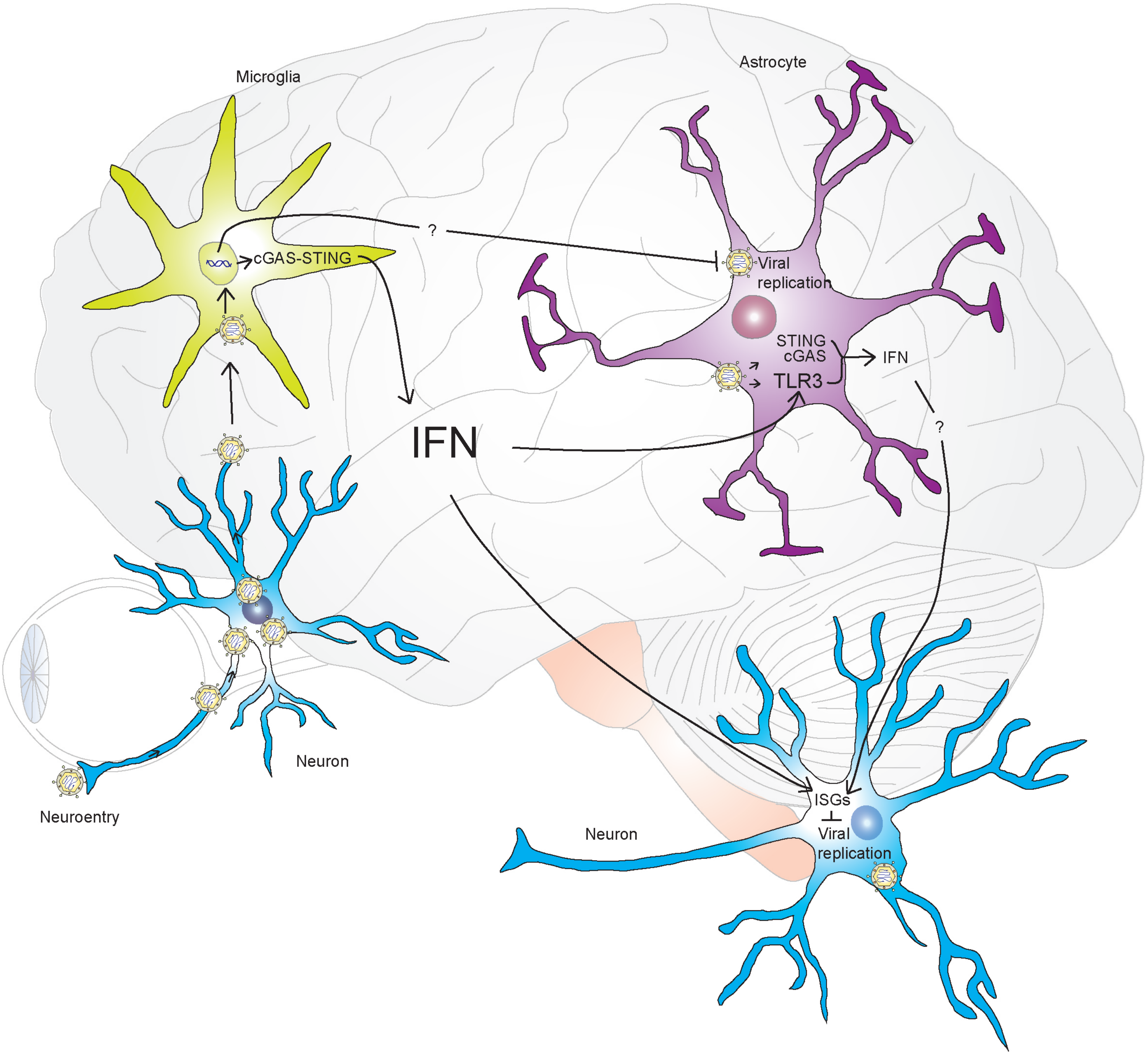

Supplementary Figure 10. From the infection in the eye, HSV-1 spreads via the trigeminal nerve to the brain stem. Within the CNS, the virus is detected by microglia through the cGAS-STING pathway. This leads to production of type I IFNs, which act on adjacent cells by binding to the IFNα/β receptor and inducing expression of IFN-stimulated genes. In neurons, this triggers an antiviral state, thus impairing HSV-1 replication and spread. In astrocytes, the microglia-mediated stimulation leads to upregulation of TLR3, and priming of this pathway, which is known to be important for control for HSV-1 in the CNS. In addition, HSV- 1 also activates a cGAS-STING-independent process in microglia, which enables paracrine transfer of antiviral activity to astrocytes. Altogether, we propose that the cGAS-STING pathway is responsible for sensing of HSV-1 by microglia in the CNS. This in turn enables microglia to orchestrate an intercellular network including both paracrine antiviral activities and priming of antiviral pathway.

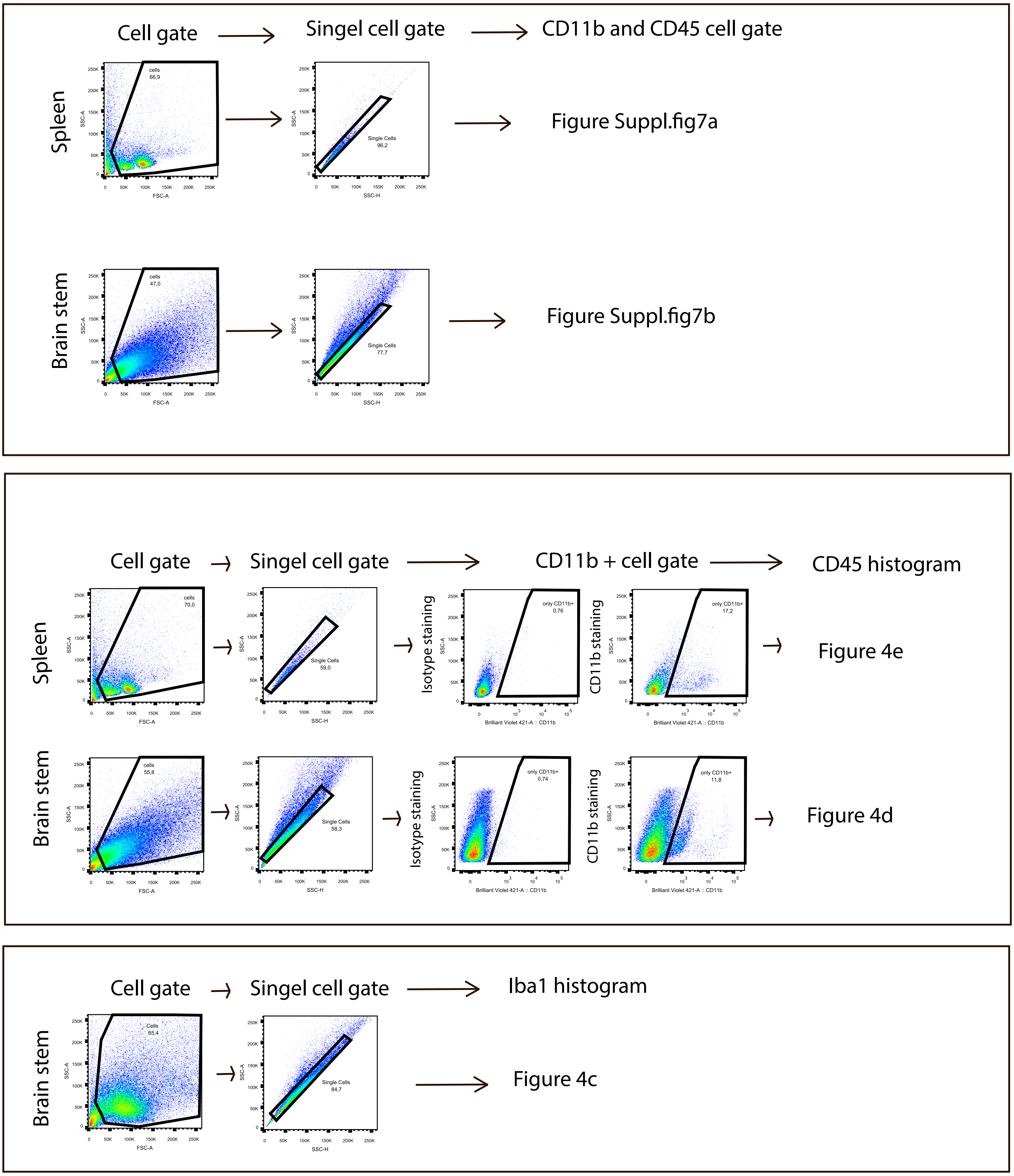

Supplementary Figure 11. The gating strategies used for the flow cytometry experiments in the present manuscript. First, a FSC/SSC-plot was made for gating all cells. The cell population was copied to a SSC-H/SSC-A-plot to identify single-cell population, which was used directly to make Suppl. fig 7a and b and Fig 4c. The single-cell population was copied to a SSC-A/CD11b-scatterplot to identify CD11b positive-cells, based on the CD11b-isotype staining. The CD11b positive- cells were then used to make the CD45 histogram in figure 4e and d. FlowJo software was used for analyzing flow data.
